# A Manycore Vision Processor for Real-Time Smart Cameras †

**DOI:** 10.3390/s21217137

**Published:** 2021-10-27

**Authors:** Bruno A. da Silva, Arthur M. Lima, Janier Arias-Garcia, Michael Huebner, Jones Yudi

**Affiliations:** 1 Automation & Control Group, University of Brasilia, Brasilia 70910-900, Brazil; eng.fis.bruno@gmail.com (B.A.d.S.); arthurlima67@yahoo.com.br (A.M.L.); 2 Graduate Program in Electrical Engineering, Department of Electronic Engineering, Federal University of Minas Gerais (UFMG), Belo Horizonte 31270-901, Brazil; janier-arias@ufmg.br; 3 Computer Engineering, Technical University Brandenburg, 03046 Brandenburg, Germany; michael.huebner@b-tu.de

**Keywords:** multi-processor system-on-chip, network-on-chip, image processing, computer vision, real-time, smart camera

## Abstract

Real-time image processing and computer vision systems are now in the mainstream of technologies enabling applications for cyber-physical systems, Internet of Things, augmented reality, and Industry 4.0. These applications bring the need for Smart Cameras for local real-time processing of images and videos. However, the massive amount of data to be processed within short deadlines cannot be handled by most commercial cameras. In this work, we show the design and implementation of a manycore vision processor architecture to be used in Smart Cameras. With massive parallelism exploration and application-specific characteristics, our architecture is composed of distributed processing elements and memories connected through a Network-on-Chip. The architecture was implemented as an FPGA overlay, focusing on optimized hardware utilization. The parameterized architecture was characterized by its hardware occupation, maximum operating frequency, and processing frame rate. Different configurations ranging from one to eighty-one processing elements were implemented and compared to several works from the literature. Using a System-on-Chip composed of an FPGA integrated into a general-purpose processor, we showcase the flexibility and efficiency of the hardware/software architecture. The results show that the proposed architecture successfully allies programmability and performance, being a suitable alternative for future Smart Cameras.

## 1. Introduction

The emergence of new trends in technology, such as the Internet of Things and Industry 4.0, pulled out several applications based on image processing and computer vision (IP/CV) techniques. Cyber-physical systems, augmented reality, and autonomous machines, among others, all have applications supported by the extensive use of cameras. Most of the conventional cameras are designed for the acquisition and transmission of images and videos. These cameras are not able to support complete applications running under real-time constraints. For these reasons, there is a need for devices capable of acquiring and processing images and videos efficiently and in real-time.

**Figure 1:**
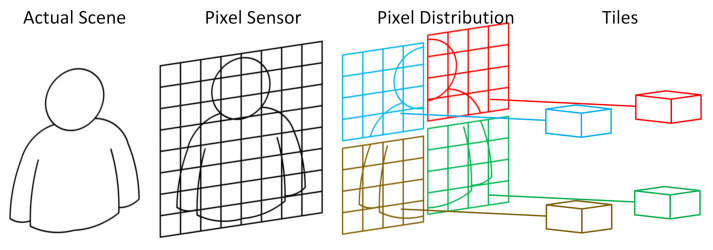
The intrinsic IP/CV parallelism explored using multiple processing tiles.

Images are inherently parallel, and the literature shows that for the IP/CV domain, the efficient parallelism exploration is the key for performance improvement. The underlying hardware/software architecture, as well as the programming model offered to the users, must be designed under application-specific constraints to reach higher performance [1,2,3]. To explore the parallelism massively, our approach was to parallelize the processing right after image capture by the pixel sensor. Figure 1 shows the concept used, in which the image is divided into parallel processing units [4].

Hardware architectures span a broad diversity: ASICs, direct field-programmable gate array (FPGA) implementations, application-specific instruction set processors (ASIPs), very-large instruction word (VLIW) processors, digital signal processors (DSPs), graphics processing units (GPUs), and multi-processor System-on-Chip (MPSoCs), etc. Each approach is an attempt to explore data parallelism, higher processing frequencies, pipelines, or complex instructions. Considering the characteristics of pixel parallelism, depicted in Figure 1, as well as in previous findings from the literature, we propose an IP/CV application-specific architecture: a manycore vision processor system. All hardware/software design decisions are based on IP/CV application-specific needs, from the processing elements microarchitecture to the Network-on-Chip (NoC) interconnects. A general-purpose processor (GPP) and a direct memory access (DMA) scheme complement the manycore to build a novel heterogeneous smart camera architecture.

The rest of this work is organized as follows: Section 2 depicts a literature review of the main challenges in the area, the state-of-art solutions, as well as the gaps we propose to cover with this work. In Section 3, we explain the HW/SW organization, the main design decisions, and implementation details of each hardware block. Section 4 explains the application used to characterize the HW/SW architecture. Section 5 presents and discusses the results. Finally, in Section 6, we present the conclusion and future works.

## 2. Related Work

In the literature, there are plenty of works towards accelerating/optimizing IP/CV algorithms, covering soft-related aspects, such as algorithm rewriting [5], to hardware-related ones, such as GPU systems [6]. This section covers recent approaches related to embedded IP/CV systems and HW/SW architectures that implement common image processing tasks.

### IP/CV Systems

As IP/CV techniques present massive data and operation parallelism, efficient parallelism exploration is potentially a solution to optimize the performance of such techniques. VLIW processors can perform several operations in parallel, offering a suitable approach. In [7], the authors merge a scalable VLIW architecture with OpenCL parallelization capabilities to build an NoC-based multi-processor for medical applications on FPGAs. They prioritize better layout and resource usage to model the network, in a data oriented approach, but lack performance for IP/CV applications compared with similar works. Similar to our work, they slice the image in multiple segments, yet demand more from the network.

The development of time-critical systems requires attention to all aspects of the HW/SW architecture. The data transmission among processors represents one of the bottlenecks in any parallel architecture. The authors of [8] implement an image processing four-port NoC architecture in Virtex II family FPGA, capable of store memory and display results at the same time. The authors of [9] present an improved six-port Torus topology architecture based on [8], each one with different functions: from acquisition to display interfaces, passing through processing units (PUs). Each core interface is composed of FIFOs instead of finite state machines (FSMs), different from our work. Even though they present an early stage prototype, there is no parallelism exploration with multitasking or image slicing. The authors separate the task into different sub-blocks and pass the whole image data through an NoC instead, which simplifies the architecture but reduces timing performance.

The authors of [10] propose an NoC for image processing algorithms. It is based on a “token-ring” approach, using one circular unidirectional network to transport commands and results data via an asynchronous network. The authors of [8,9,10] explore the scalability of NoC communication, and each PU is responsible for specific parts of the desired IP/CV algorithm, differing mostly by the six-port Torus and token-ring network types. This approach favors local optimization of each PU; however, it does not explore the operation parallelism of the IP/CV data, which leads to the need for much higher operating frequencies. Our architecture organization also explores the NoC scalability features, but furthermore, by the exploration of pixel-level parallelism, we can operate at lower frequencies, which could represent smaller power consumption.

The authors of [11] present a multi-processor architecture based on a Spidergon NoC topology. It is a heterogeneous processing system, where each tile has its specialization: memory, general-purpose processor (GPP), and motion estimation, etc. Each tile can reach another one through the NoC, and several different IP/CV algorithms can be implemented using all or some of the tiles. There is some task-level parallelism, since the processing tiles can work in parallel, and high throughput is reached. That work could explore the pixel-level parallelism; however, the authors did not consider that possibility.

An NoC-based MPSoC is explored for some IP/CV algorithms in [12]. The authors use an MPI (message passing interface) to parallelize the algorithms and distribute the threads among the cores. It enables multi-processing with high-level abstraction in tightly constrained devices and, similar to our work, divides the image into slices and distributes them inside the processor’s memories. Our approach is also based on an NoC-based MPSoC; however, we have all cores executing the same program in parallel, but over different data sets (pixel regions), instead of the MPI model. We also use an FPG-based architecture instead of an ASIC, such as [12] does.

Another relevant architecture type for IP/CV implementations is the GPUs. A GPU is commonly able to explore vector operations efficiently. Several IP/CV algorithms can be expressed in such a way that the compilers are effective in addressing the instruction-level parallelism. In [6,13], a soft-GPU architecture is presented. Similarly to our approach, the authors developed an FPGA overlay exploring the platform features (DSP blocks, distributed memory, logic blocks, and interconnects) to optimize the architecture design, showing the feasibility of FPGA overlays as an end-user platform. It develops an FGPA GPU (called FGPU) architecture with computing units, a basic processing block of the FGPU architecture with eight custom processing elements to perform SIMD instructions. It implements IP algorithms such as Sobel, compass edge detector, and filter algorithms for benchmarks. Different from our work, the authors develop a general purpose graphics processing unit for FPGAs, focusing on high-performance gain and power consumption, instead of an IP/CV specific solution.

The NVidia Tegra Tx1 is a heterogeneous architecture composed of an embedded GPU architecture with 256 CUDA cores and a quad-core ARM processor. In [14], that platform is used to develop a smart industrial camera able to perform real-time object recognition. The authors show that an embedded GPU can efficiently explore the parallelism in IP/CV algorithms and provide high throughput and flexibility.

A general-purpose pixel distributor for parallel processing in FPGAs is depicted in [15]. The authors address the importance of such architecture for real-time image processing and the demand for an efficient parallel distribution system to reduce the required memory. Stream processing is the primary approach of their work, with specific processing units directly implemented in FPGA, and also exploring the pixel-level parallelism. However, despite stream processors, tiny processing elements based on RISC architectures are used, favoring flexibility for any IP/CV application.

Some authors explore the FPGA dynamic reconfiguration to improve online flexibility [16]. However, the reconfiguration time is far too time-consuming to be used in real-time applications. In our architecture, full flexibility is provided by the use of soft-programmable processing elements.

The authors of [1,17,18] propose a methodology for the design and programming of next-generation manycore vision processors. The authors suggest new design architectures that optimize multi-cost functions: memory and resource usage, communication cost, power consumption, and hardware speed. Their work computes microarchitectures for the IP/CV applications that explore pixel operation parallelism using multiple iterations and SystemC/TLM models to make better design decisions for this kind of application. In addition, they analyze multiple parallelism aspects in manycore vision processors based on algorithm characteristics, pixel-level, and multiple stages of design space. They explore various subsystems in the architecture to extract better parallelism, develop a simulation tool using SystemC/TLM 2.0, and show possible architectural choices to improve this kind of processing system. Furthermore, they execute the canny edge detector as a reference algorithm, which computes the number of operations and memory accesses necessary to the system. The authors build the architecture for 16x16 pixels per tile prototype in FPGA, showing that it is viable compared with other state-of-the-art canny edge detector (CED) implementations. In conclusion, they obtain a manycore architecture based on simulations in SystemC/TLM 2.0 and other design space exploration techniques. Our work concerns practical implementation issues based on their results to build a viable manycore vision processor on FPGAs based on their findings.

The authors of [19] present a high-level synthesis tool to facilitate a time-to-market heterogeneous MPSoCs design. The hardware architecture combines Microblaze softcore flexibility with HLS practicality to implement multiple designs for different project constraints. Despite the MPSoC implementation in FPGA using the Network-on-Chip, our approach focuses on lower-level aspects instead of the programming model and high-level synthesis. The choice from [19] reduces the design effort but has performance reduction compared to more specialized architectures such as ours for IP/CV applications.

The authors of [20] set up a method to program heterogeneous MPSoCs using the Xilinx SDSoC framework and other open-source tools. The application profiles automatic instrumentation of the code to the designer, which makes better decisions if necessary. This approach differs from our work because of its focus on a high-level tool to ease rapid prototyping in manycore systems. We provide design choices for homogeneous MPSoCs in overlay architectures, implementing the same task for all cores.

Table 1 shows a comparison of different architectures cited in this section with our work in terms of processing element type, communication structure, programmability, and main features. Most of the work use NoC for communication, and have programmable devices. This shows the interest of recent IP/CV related architecture with programmability and high-level design, using OpenCL, MPI and Vivado HLS in many of them as a possible way to improve performance and time to market. We use the term *programmability* as the characteristic of an architecture being *application-agnostic*. This means that, once the underlying hardware is defined, any application in the IP/CV domain can be implemented. As a result, even though FPGAs are field-programmable, to change the application, it is necessary to redesign the architecture.

The next session explains the HW/SW platform used to develop the complete Smart Camera concept.

## 3. The HW/SW Platform

**Figure 2:**
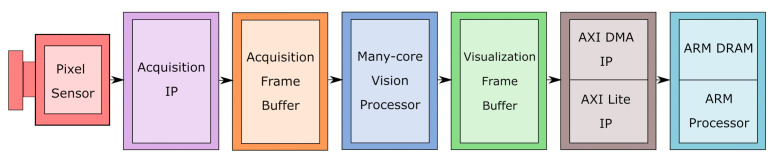
Block diagram of the complete Smart Camera system.

The architecture proposed in this work was designed considering the diversity and complexity of IP/CV algorithms, the parallelism exploration on different abstraction levels, and the hardware costs. Figure 2 shows the block diagram of the complete Smart Camera proposed [21]. We selected a ZYNQ Ultrascale+ device (ZCU104 development kit from Xilinx), a state-of-art SoC [22], which integrates a GPP with an FPGA fabric in the same chip. The first block is a common pixel sensor module, which connects to the development board through a GPIO port. The ZYNQ Chip receives the pixel stream and stores the input image in the acquisition frame buffer. The pixels are then distributed among the tiles within the manycore vision processor to be processed. After the image processing, the output image is stored in the visualization frame buffer, which is then read out through DMA by an ARM processor, integrated into the SoC. The next sections explain in detail all the Smart Camera components.

### 3.1. Pixel Sensor

**Figure 3:**
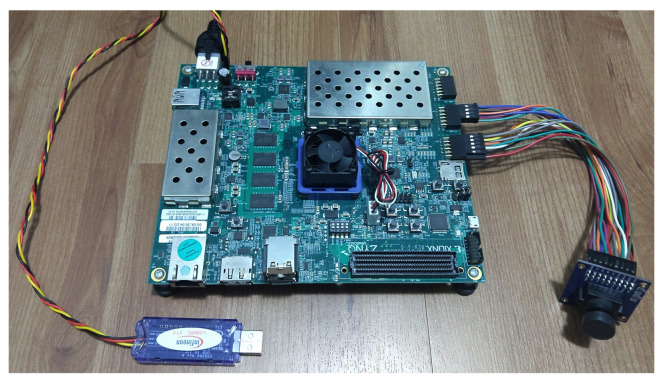
Photography of the Xilinx ZCU104 development kit with the OV7670 CMOS sensor and the Infineon power monitor device.

This work uses the OmniVision OV7670 CMOS sensor [23], a commercial OEM model with industry standard parallel interface. The sensor is set to QVGA resolution, 320 × 240 pixels, achieving up to 60 frames per second in the current setting. It outputs pixels in RGB444 encoding, which is therefore converted to grayscale in the Acquisition IP. The sensor pins are connected directly to the ZCU104 board through two GPIO interfaces, as shown in Figure 3 [21].

### 3.2. Acquisition IP and Acquisition Frame Buffer

The Acquisition IP is a group of different modules: to control the CMOS sensor, to receive pixel data, and to store it in the acquisition frame buffer. This scheme uses an open-source project [24] as the main reference with modifications.

Five modules, i.e., AXI Camera Control IP, Debounce, OV7670 Capture, RGB444 to Grayscale and OV7670 Controller, compose the IP, either for control or data sampling. OV7670 Controller module is responsible for the CMOS initialization. The controller sets the image size, output data format (RGB444 in this case), prescaler, contrast, gamma, UV auto adjust, image orientation, color conversion, VSYNC/HREF setups, and other configurations.

The sensor could be configured to output YUV format, avoiding the use of RGB to grayscale conversion; however, we wanted to show that it is also possible to explore direct FPGA IP/CV implementations, such as the ones depicted in Section 2.

### 3.3. Manycore Vision Processor

**Figure 4:**
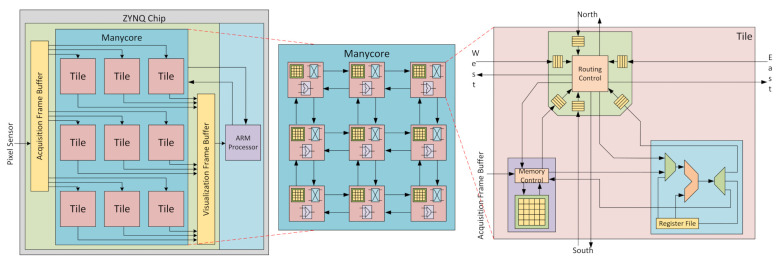
The manycore hardware architecture designed in our work.

This section describes the manycore vision processor architecture and its HW/SW integration, as shown in Figure 4 [4]. It is made of basic units, called tiles, that process and store pixel values in memory and communicate with the other tiles using a router. Multiple tiles form a 2D-mesh Network-on-Chip, which transmits pixel data and control messages, forming a homogeneous manycore processing system. We run the same program code in all tiles to explore natural pixel parallelism, providing the usability at the IP/CV domain.

#### 3.3.1. Pixel Memory

**Figure 5:**
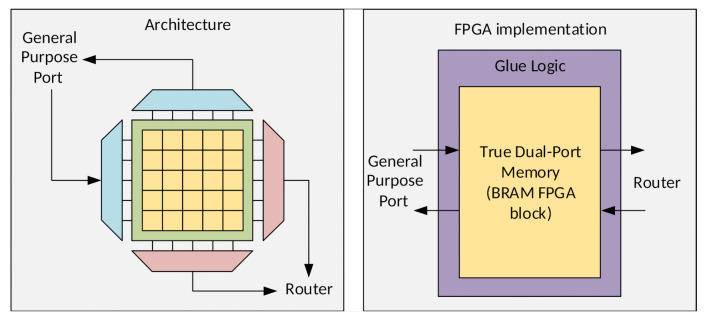
The pixel memory architecture.

The PM has to interface with the router, the PE, and also interact with external components such as the ARM processor or the input/output image buffers, as shown in Figure 5 [21]. Each pixel memory ideally stores a defined image region. In practice, there are some exceptions in border tiles, where it can have more storage than pixels in its sub-image. This happens because pixels can not be equally divided in regions depending on the manycore size. Furthermore, our automation tool has limitations and does not optimize the image slice for multiple architectures, which is not possible depending on the image resolution and MCVP size. However, for simplicity, these exceeding addresses are not taken into account in the implemented algorithms. Furthermore, Vivado’s block memory code generator cannot cut the exact BRAM slices depending on the architecture and image configurations due to its physical limitations, but it does not affect the implementations.

#### 3.3.2. Processing Element

**Figure 6:**
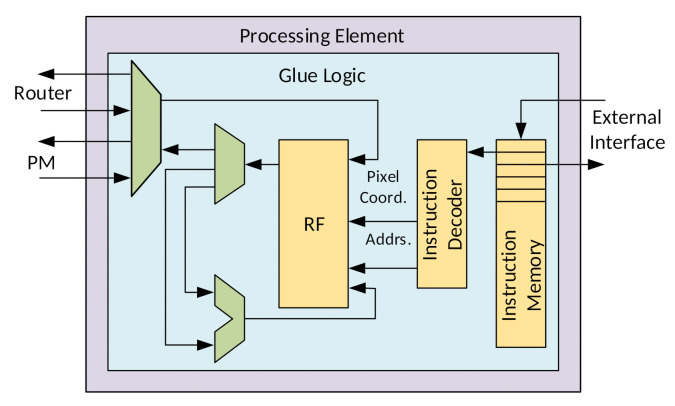
Processing element microarchitecture for P-type and R-type instructions.

Processing elements must be able to perform simple computations such as basic arithmetic and branch operations. Furthermore, they must be compliant with the application-specific needs of IP/CV algorithms. With that in mind, a minimalist RISC processor, with only 16 instructions, was designed, as shown in Figure 6 [21]. The PEs have access to the register file and to the PM to use in tasks. Instruction memories store programming code for execution.

The PE was developed focusing on: basic arithmetic operations necessary to most image processing algorithms, defined here as *R-type instructions*; branch and jump operations to control flow, named as *Branch* and *Jump instructions*, respectively; pixel data communication (*P-type*) and *Control instructions*. Those five constitute all *instruction formats* necessary for the PE to implement any type of IP/CV algorithm, turning the PE in a Turing-complete machine.

#### 3.3.3. Router

**Figure 7:**
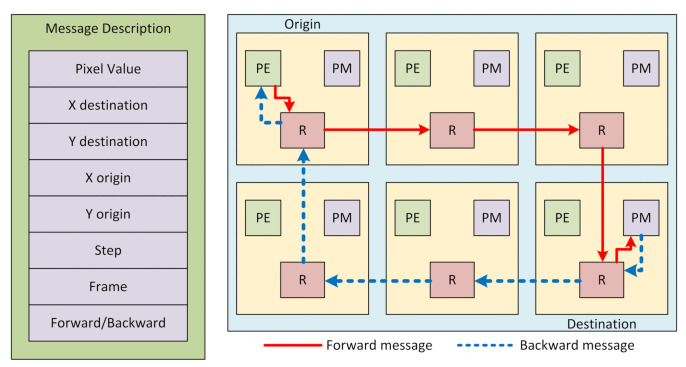
Message description and route.

The router is the component responsible for exchanging pixels among the tiles. Its interface consists of 6 ports, each one with one input and one output channel. All the channels are unidirectionally connected to the neighboring tiles (N, S, E, W) and the local PE and PM. Between each tile pair, there are message buffers used to avoid network stalls. The size of these buffers is configurable and depends on the algorithms used and the overall architecture configuration (image resolution and number of tiles). Figure 7 illustrates a message and how it is routed through the manycore [21].

For example, if a PE has to perform a get-pixel instruction (GPX), which means that it needs a specific pixel at the RF to process, the PE wrapper verifies if the pixel belongs to its image region or another one. If it is a local pixel, the wrapper asks the local PM for the pixel value. In the case that a pixel is in another image region, the wrapper asks the local router. The router has an arbiter and, when it is the PE communication slot, the router decodes the destination and forwards the message to a neighbor router. Take Figure 7 as a sample, the top left tile requests a pixel that belongs to the bottom right one. Because of this distribution, the forward message passes through all routers in the red arrow route until it arrives at its destination. After obtaining the desired pixel, the PM sends it to the nearest router, which passes the message (as shown by the dashed blue arrow) to neighbor routers until the pixel arrives at its origin.

### 3.4. Visualization Frame Buffer

The visualization frame buffer controls pixel transfer from the manycore vision processor to the ARM processor, after the MCVP ends the processing of a frame. It has one AXI4-Lite interface to control and read relevant data from visualization FB. Furthermore, a memory interface, made of a true dual-port block RAM with two write ports in read-first mode [25], stores pixels written by the MCVP. This IP deals with two interfaces to link the manycore with the ARM processor: a memory interface from the MCVP, and an AXI4 Stream interface to communicate with the AXI DMA IP.

An AXI4 Stream interface sends data without ARM direct intervention. This approach reduces the processor load but demands a new IP to convert the stream to memory-mapped transfer (AXI DMA IP) and a finite state machine to read pixels from the block RAM and send it through AXI Stream interface.

### 3.5. AXI Direct Memory Access IP

This work uses an AXI direct memory access (DMA) IP [26] to write inside ARM’s DRAM. The work uses this model to deal with the DMA to reduce ARM’s workload. Another way to use it is to interrupt the processor every time a DMA iteration is complete. Them, the IP needs to be reprogrammed and so forth. Using descriptors, there is no need to interrupt the processor and reprogram anything, although it is still possible to interrupt the processor if necessary.

In the implemented design, one memory segment is reserved for the image, but the architecture can handle even multichannel cases. This situation can deal with two distinct cameras for stereo computer vision, for example.

### 3.6. ARM Processor

This work uses a Zynq UltraScale+ XCZU7EV-2FFVC1156 MPSoC with an ARM Cortex-A53-based application processing unit (APU) from the ZCU104 development kit. In this work, the ARM processor runs a bare-metal implementation, using only a single core from the four available. The ARM has PL-PS interruption enabled to address DMA IP needs. Furthermore, the initialization process and other low-level tools are all dealt with by Xilinx proprietary tools: the Vivado Design Suite to synthesize and implement the architecture, and the Xilinx Software Development Kit to handle software and FPGA setup.

The ARM processor is used to configure, program, monitor and debug the manycore system, as well as to contribute to the IP/CV application in higher abstraction levels. It communicates to the manycore system through the AXI (Lite/DMA) interfaces described earlier in the text. Through these interfaces, the ARM processor has access to pixel memories (PMs), instruction memories (IMs), and special-purpose registers for control and debug purposes.

## 4. Application Domain Analysis

The architecture proposed in this work aims to implement any type of IP/CV applications. In this context, we decided to show its flexibility with the implementation of an application suitable to explore different types of IP/CV algorithms: a motion estimation using the Harris corner detector (HCD). Several IP/CV algorithms manipulate pixels in similar ways. The Khronos group consortium established the OpenVX standard as a set of IP/CV functions selected as the most representative components of more complex applications.

In Table 2, we classify all 58 OpenVX functions into five classes, depending on how the image pixels are analyzed/manipulated [27]. In the first class, there are some complex applications, which can be built by combining functions from other classes. The last three classes are the lower-level ones and responsible for direct pixel manipulation. These classes are the most computationally expensive, and efficiently explored by our architecture. To show the performance of our solution for the IP/CV application domain, we selected an application from the first class, which we consider as representative of the most common applications in this domain: a motion estimation based on the Harris corner detector.

### 4.1. Harris Corner Detector

**Figure 8:**
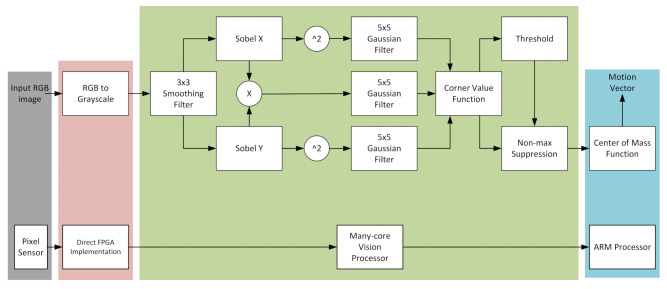
Motion estimation using a corner detector.

Figure 8 shows the HCD processing chain based on common textbook implementations [28]. The HCD algorithm is composed of several smaller blocks, all of them present in the OpenVX specification. To have different comparisons to the literature, we evaluated the performance of the complete HCD, the convolution with sizes 3 × 3, 5 × 5, 7 × 7, and the Sobel edge detector.

The HCD was implemented completely in the manycore architecture. It reads the grayscale image from the acquisition frame buffer, processes it with the HCD algorithm, and outputs the resulting image to the visualization frame buffer.

### 4.2. Motion Estimation

The ARM processor is responsible for the motion estimation. It receives the manycore’s processed images (HCD results) through the DMA interface. For each image, the center of mass (CoM) of the detected corners is computed in a software routine. A CoM’s displacement vector is then determined containing the CoM’s trajectory. The motion estimation described here is a global operation, in the sense that it requires the corners positions of the complete image. This level of abstraction is better implemented in the ARM processor than in the manycore.

## 5. Results and Analysis

This section presents the results of the complete IP/CV processing chain, from the image acquisition in the OV7670 sensor to the ARM’s motion estimation. All data use the QVGA image resolution with the FPGA running at 100 MHz, and the ARM processor running at 667 MHz. We intend to address the general characteristics of the manycore, discussing its advantages and disadvantages. Figure 3 shows a photograph of the OV7670 CMOS sensor and Infineon power monitor device attached to the ZCU104 Development Kit used in this section’s results.

### 5.1. Resource Usage

Table 3 shows the hardware resources used by the complete chain, for different manycore sizes from 1 to 81 Tiles, which reaches the physical limit of the Xilinx ZCU104 Evaluation Kit (currently a state-of-the-art device) [4]. We can see in the table the manycore’s scalability while increasing the processing parallelism levels.

### 5.2. Performance Evaluation

The performance experiment was set to capture 500 frames, where the camera is set to a free-capture mode, saving all the frames in the acquisition frame buffer. The ARM processor controls the MPSoC to wait for the camera VSYNC signal and guarantee correct frame processing. As image transfer time from the acquisition FB to PMs takes about 1 ms, which is similar to the DMA transfer time, there is no pixel loss in free-capture with the camera producing 60 fps.

To determine the processing delay, the timer starts to count when the ARM finishes the setup and initialize peripherals (the MCVP, DMA, camera, and Acquisition FB) and stops to count after writing the last pixel in ARM’s DRAM through the DMA.

**Figure 9:**
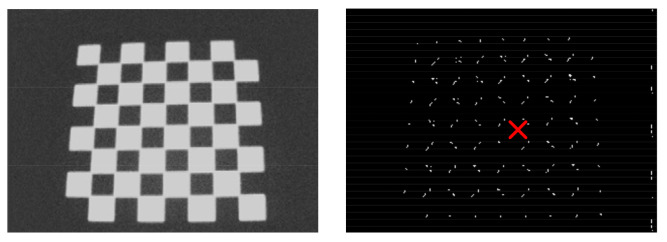
**Left**: real image acquired by the pixel sensor; **right**: center of mass (red cross) calculated by the ARM processor over an HCD output from the manycore.

Five algorithms are implemented: the Harris, Sobel, 3 × 3, 5 × 5, and 7 × 7 convolutions. Figure 9 (left) shows a chessboard captured image from the sensor and read from ARM DRAM through an UART interface, for evaluation purposes. Figure 9 (right) depicts the final CoM computed over an HCD output image.

**Figure 10:**
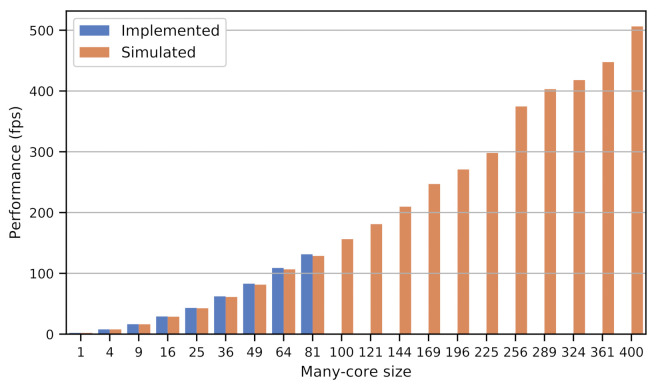
Performance in frames per second comparing implemented and simulated results for the Harris Detector.

Figure 10 shows the performance of the manycore system in simulation (up to 400 tiles) and implemented alone in the FPGA-fabric (up to 81 tiles) [4]. The implementation could reach only 81 tiles, due to the device physical limitations. However, through simulation, we can see the scalability and performance improvement by exploring the natural parallelism of the IP/CV algorithms.

**Figure 11:**
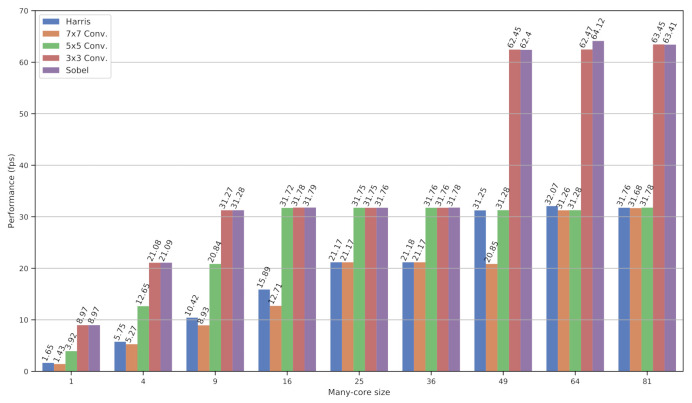
Execution time for multiple algorithms with acquisition and visualization scheme.

Figure 11 shows time performance results for the algorithms executed, from 1 to 81 PEs, in frames per second (*fps*) [4]. The results differ from Figure 10 due to the synchronization with the image sensor. The sensor configuration could only be used to output single-shot images (1 and 4 tiles), 30 fps (9 to 36 tiles), and 60 fps (49 to 81 tiles). The processing architecture is able to reach the performance depicted in Figure 10; however, the pixel sensor was the system’s main bottleneck. It is important to highlight that the processing architecture was designed to fulfill the real-time constraints given by the sensor frame rate.

It is not easy to compare different architecture types, as well as systems designed with different VLSI technologies. To have a kind of normalization, we computed the **cycles/pixel**, which is a metric that shows how efficiently a computing architecture implements an IP/CV application. This metric is independent of the VLSI technology and the operating frequency. We believe that, with this normalization, we can fairly compare the architectural designs from several years ago until now.

A comparison, only for the Sobel edge detector against our 81 tiles implementation, is shown in Table 4 [4]. We can see that our architecture provides a good performance, while still being flexible. The authors of [20] build a method to program heterogeneous MPSoCs using the Xilinx SDSoC framework. It implements an edge detection algorithm in multiple scenarios: software only, HW/SW with static and runtime task mapping/scheduling. The authors of [12] uses a 2D mesh NoC-based 16 RISC core processor to implement different image processing tasks for its parallel programming model called threaded MPI. The authors of [19] develop a high-level synthesis tool to facilitate time-to-market heterogeneous MPSoCs design, using an MPI-based programming model and Vivado tools for HLS and TCL scripting.

Table 5 compares our best timing performance MPSoC with related architectures for the Harris corner detector application [4]. We show in the table two results: the simulated one, with 400 tiles, and the implemented one (limited by the FPGA size), with 81 tiles. The authors of [29] utilize a Jetson (ARM and GPU) similar to this work, with a GPU instead of using programmable logic. The authors of [30] implement HCD on a ASIC SIMD architecture. The authors of [12] use an NoC-based MPSoC with 16 RISC cores assisted by an external ARM CPU. The authors of [31] build the application in a processor array using VLIW PEs and point-to-point communication, building a tightly-coupled processor array. The authors of [32,33,34,35] implement pipelined HCD architectures in FPGA.

The motion estimation was implemented only in the ARM processor. Its performance is independent of the manycore’s performance, for the same input image. The center of mass computation and the motion estimation resulted in an average performance of only 1.43 ms.

In general, non-programmable pipelined FPGA architectures have better results but lack flexibility. However, other device types can also achieve similar performance, in terms of frames per second, for ICs that can run at higher frequencies but consume more power. It is important to emphasize that this work explored the platform physical limits in the Zynq ZCU104 board and still can reduce HCD execution time for larger FPGAs. Moreover, our solution brings full flexibility with a performance close to non-programmable architectures. Reference [12] is also an NoC-based MPSoC with RISC processors, and the most similar implementation to our architecture in Table 5.

The main goal of our architecture is to provide a feasible processing system for real-time IP/CV applications. A very popular architecture with similar utilization is a GPU, for example, the NVidia Jetson family [29]. Despite not being similar in the microarchitecture characteristics, we can compare our work to GPUs, considering the huge number of processing cores. We divided the comparison with GPUs into two parts: the first one with a soft GPU, and the second one with a commercial GPU.

**Figure 12:**
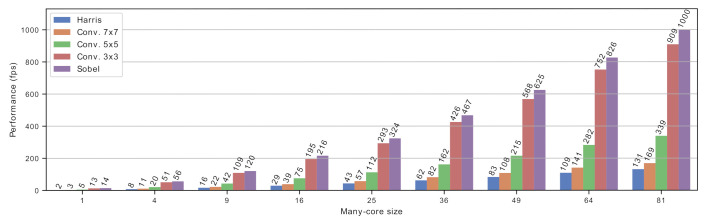
Performance in frames per second per architecture, all running in a testbench without a camera.

Our manycore was implemented as an overlay architecture, so it should be fair to compare it with an overlay GPU. In [6], the authors provide the FGPU, a general-purpose GPU optimized for FPGAs. The FGPU architecture is composed of several computing units (CUs), where each CU has eight processing elements. The FGPU’s programming model is based on single-instruction multiple-threads (SIMT) and provides full-thread divergence, which means that each PE operates individually over the same program. Our manycore has the same characteristic, where each PE is independent and runs the same program. The authors in [6] benchmarked the FGPU with a varied number of CUs, as well as with a diversity of application domains. The authors connected the FGPU to an ARM processor using the AXI bus in a similar fashion to our work. The ARM was then responsible for sending data to be processed and receiving back the results. To perform this comparison, we used the ARM processor to operate in the place of our camera, resulting in a very similar testbench. Figure 12 shows the results for our testbench in different configurations, running some applications [4].

In [6], there is the benchmarking of a *Sharpen* filter, a convolution operation in a 5×5 neighborhood. For the sake of comparison, we selected their results with an eight-CUs configuration (64 PEs), the one with the highest number of PEs. Table 6 shows the comparison in its first two rows. We can see there that the manycore architecture is more efficient in parallelism exploration for the same number of PEs, reaching a higher throughput (pixels/s) with a smaller operating frequency.

The second comparison is against a commercial embedded GPU [36]. The third and fourth rows of Table 6 show the comparison. In [36], a NVidia Jetson TX2 with 256 cores runs the HCD algorithm. We compared it with our manycore with 64 PEs (4 times fewer PEs than the Jetson TX2). We can see in Table 6 that our architecture is more efficient in the parallelism exploration, with 5× fewer cycles needed per pixel.

### 5.3. Power Consumption

The ZCU104’s power system has components to monitor voltage and current on the power rails by the Infineon manufacturer [37]. An Infineon USB005 USB cable [38] is used to obtain power results directly from a board’s specific connector.

We used the PowIRCenter GUI application to obtain power results from all rails. Considering that the ZCU104 development kit shares different peripherals in the same rail, mixing the FPGA fabric with DRAM power supply [22], for example, it is not possible to separate FPGA from ARM or other subsystems. For this reason, experimental power results concern the total instantaneous consumption in Watts (*W*) for all rails, including the pixel sensor. This means that the power measured is bigger than the power used by the Sensor + FPGA + ARM part.

Similarly to previous timing results, this subsection also tests the Harris, Sobel, 3 × 3, 5 × 5, and 7 × 7 convolutions, varying the number of processing frames with the algorithm and manycore size. For each algorithm, a sequence of approximately 500 images was captured to provide an average power consumption.

**Figure 13:**
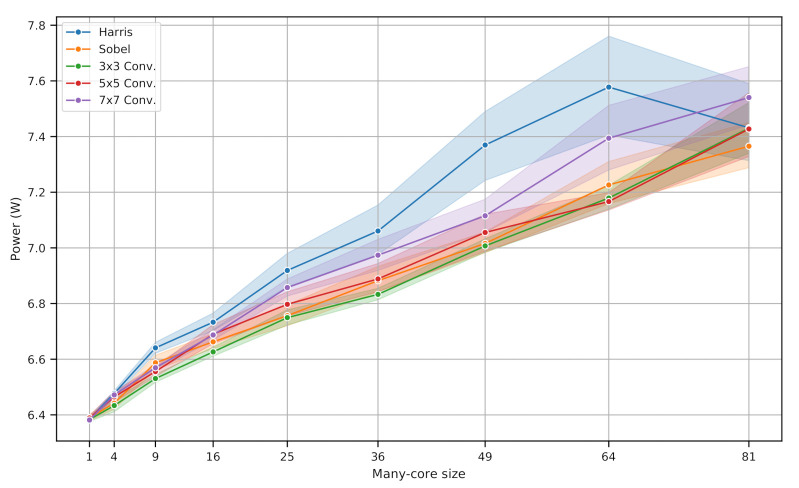
Power consumed for different algorithms and architecture sizes.

Figure 13 shows the power performance graphic for different algorithms and architecture sizes. Lines and points represent the measurements mean, and the shaded area is the 95% confidence interval based on the mean estimator [39]. The power behavior relates to all of the architecture components: the pixel sensor, image capture system, DMA, manycore, ARM processor, and other board subsystems.

With more tiles, there is more instantaneous power consumption because there are more pixel transmissions between each of them. With more routers and memory in use, the data transferences between each tile and routers’ queues occupation is less predictable. Furthermore, as we deal with a large digital system, in terms of resource usage that consumes thousands of logic units, the Flip-Flops clock distribution is not completely uniform, causing propagation delays. Combining the MCVP behavior with the FPGA and physical characteristics, the system has variable and less predictable instantaneous power consumption, leading to a standard deviation increase. As expected, the HCD takes more power to be finished since it has more operations that also take more time.

## 6. Conclusions

In this work, we developed a Smart Camera system based on a manycore architecture for real-time image processing and computer vision applications. The main contribution of this work is a novel manycore architecture specially designed for the IP/CV application domain. All design decisions were made considering the domain-specific needs, which resulted in a refined and efficient hardware/software architecture.

Our approach makes use of *Region-based* and operation level parallelism to optimize the processing time. Additionally, we designed a pixel distribution and control unit using an embedded ARM processor and AXI bus scheme. All subsystems are addressed and explained with construction details, focusing on matching application-specific needs. As a proof-of-concept, we implemented some IP/CV algorithms: the motion estimation, Harris corner detector, Sobel edge detector, and convolution filters. The results show that our architecture is capable of overcoming similar real-time architecture depending on the manycore size and application demands. The architecture is also flexible and easy to scale for higher numbers of tiles.

The manycore architecture proposed was compared to state-of-art approaches based on highly specialized FPGA implementations, reaching a good performance, while providing an application-agnostic solution. In comparison to embedded GPUs (commercial and academic ones), our solution is more efficient on parallelism exploration. GPUs are directly programmed in C/C++. Our architecture does not yet have a C/C++ compiler; however, it is almost as programmable as the GPU solutions.

In future works, we envision the utilization of a higher frame rate sensor to reach the full potential of the architecture. The proposed system is heterogeneous: we can implement the IP/CV algorithms in different architectures (direct FPGA, manycore, ARM processor). A mapping tool to obtain the algorithm description and divide it optimally over the system would also be beneficial. In addition, the other cores of the ARM processor could also be used to perform control and processing separately, as well as to run an embedded Linux operating system, bringing even more application possibilities to our Smart Camera system.

## Figures and Tables

**Table 1:** IP/CV reference comparison.

Reference	PE Type	Communication	Application Agnostic	Main Feature
[7]	VLIW	NoC	yes	FPGA overlay architecture to explore PS-PL
[8,9]	Heterogeneous	NoC	no	Early NoC proposal for IP/CV applications
[10]	Heterogeneous	NoC	no	Multiple Processing Units in asynchronous NoC
[12]	RISC	NoC	yes	Proprietary MPSoC and use of MPI model
[6]	Soft-GPU	Bus	yes	FPGA overlay resources optimization, use of soft-GPUs
[14]	GPU	Bus	yes	Achieve real-time object recognition with GPU/CPU combination
[1]	RISC	NoC	yes	Spatial parallelism exploration achieves high performance scaling
[19]	RISC	NoC	yes	HLS synthesis tool for a heterogeneous system built for FPGAs
[20]	Heterogeneous	Bus	no	Method to explore HW/SW design choices with few user interaction
Our work	RISC	NoC	yes	Overlay architecture that explores spatial parallelism with region-based processing

**Table 2:** Classification of the 58 OpenVX functions by operation structure.

OpenVX Function	Classification
Canny Edge Detector, Fast Corners, Gaussian Image Pyramid, Histogram of Oriented Gradients, Harris Corners, Hough Lines Probabilistic, Laplacian Image Pyramid, Optical Flow Pyramid, Reconstruction from a Laplacian Image Pyramid, Equalize Histogram	composition of different types
Mean and Standard Deviation, Min, Max Location, Tensor Add, Tensor Convert Bit-Depth, Tensor Matrix Multiply, Tensor Multiply, Tensor Subtract, Tensor Table LookUp, Tensor Transpose, Control Flow, Data Object Copy, Histogram of Oriented Gradients, LBP descriptors	non direct image operation
Absolute Difference, Arithmetic Addition, Arithmetic Subtraction, Bitwise AND, Bitwise Exclusive OR, Bitwise Inclusive OR, Bitwise NOT, Channel Combine, Channel Extract, Color Convert, Convert Bit Depth, Magnitude, Phase, Pixel-wise Multiplication, Remap, Table Lookup, Thresholding, Weighted Average	Pixel to Pixel
Bilateral Filter, Box Filter, Custom Convolution, Dilate Image, Erode Image, Gaussian Filter, Integral Image, Match Template, Max, Median Filter, Min, Non Linear Filter, Non-Maxima Suppression, Sobel 3 × 3	Region to Pixel
Scale Image, Warp Affine, Warp Perspective	Region to Region

**Table 3:** Resources for the complete IP/CV processing chain, QVGA image, different manycore’s sizes.

	Device	1 Tile	4 Tiles	9 Tiles	16 Tiles
PL Max. Freq. (MHz)	-	134.77	128.85	122.80	122.88
LUT	230,400	9841	16,057	26,117	39,765
LUTRAM	101,760	789	1557	2837	4629
FF	460,800	9733	15,219	24,164	36,843
BRAM	312	195	197	198	205
URAM	96	1	4	9	16
DSP Blocks	1728	6	18	38	66
	25 Tiles	36 Tiles	49 Tiles	64 Tiles	81 Tiles
PL Max. Freq. (MHz)	120.61	122.01	122.56	122.70	111.42
LUT	56,837	80,944	110,941	141,772	178,582
LUTRAM	6933	9749	13,077	16,917	21,269
FF	53,240	73,421	97,350	124,997	156,400
BRAM	195	219	234	237	207
URAM	25	36	49	64	81
DSP Blocks	102	146	198	258	326

**Table 4:** Comparison of implementations of Sobel edge detector.

Reference	Architecture	Cycles/Pixel	Application Agnostic?
[20] 2019 *	MPSoC-FPGA	1.06	no
Our (impl.)	MPSoC-FPGA	1.52	yes
[12] 2015	MPSoC-ASIC	2.67	yes
[19] 2019	MPSoC-FPGA	64.13	yes

* Includes the initial image transfer time.

**Table 5:** Comparison of implementations of the HCD.

Reference	Architecture	Cycles/Pixel	Application Agnostic?
[35] 2014 **	FPGA	1.00	no
[34] 2014	FPGA	1.00	no
[31] 2013	FPGA	1.36	yes
[33] 2017 **	FPGA	2.11	no
Ours (sim.)	MPSoC-FPGA	3.02	yes
[32] 2013 **	FPGA	3.03	no
[30] 2010	SIMD ASIC	3.42	yes
[12] 2015	MPSoC-ASIC	7.07	yes
[29] 2018 *	Embedded GPU	7.19	yes
Ours (impl.)	MPSoC-FPGA	11.61	yes

* Includes the initial image transfer time. ** includes acquisition and visualization scheme.

**Table 6:** Efficiency comparison among.

Architecture	Algorithm	Image Resolution	Time Per Frame	fps	freq. (MHz)	Pixels/s	Cycles Per Pixel
MCVP-64 (64 PEs)	Convolution 5 × 5	256 × 256	3.55	282	100	18,481,152	5.411
FGPU-8 (64 PEs) [6]	Sharpen 5 × 5	512 × 512	6.1	16.4	250	4,299,162	58.150
MCVP-81 (81 PEs)	HCD	256 × 256	9.17	109	100	7,143,424	14
NVidia Jetson TX2 [36] (256 cores)	HCD	640 × 480	26	38	854	11,673,600	73.156

## Data Availability

Not applicable.
